# Primary antimicrobial resistance among Mycobacterium tuberculosis isolates from HIV seropositive and HIV seronegative patients in Dar es Salaam Tanzania

**DOI:** 10.1186/1756-0500-1-58

**Published:** 2008-07-31

**Authors:** Willy Urassa, Ferdinand Mugusi, Eduardo Villamor, Gernard Msamanga, Candida Moshiro, Ronald Bosch, Elmar Saathoff, Wafaie Fawzi

**Affiliations:** 1 Departments of Microbiology and Immunology, Muhimbili University of Health and Allied Sciences, Dar es Salaam, Tanzania; 2 Internal Medicine, Muhimbili University of Health and Allied Sciences, Dar es Salaam, Tanzania; 3 Community Health, Muhimbili University of Health and Allied Sciences, Dar es Salaam, Tanzania; 4 Behavioural Sciences and Biostatistics, Muhimbili University of Health and Allied Sciences, Dar es Salaam, Tanzania; 5 Department of Infectious Diseases and Tropical Medicine, Leopoldstr, Munich, Germany; 6 Departments of Nutrition and Epidemiology, Harvard School of Public Health, Boston, MA, USA; 7 Biostatistics, Harvard School of Public Health, Boston, MA, USA

## Abstract

**Background:**

The United Republic of Tanzania is one of the 22 high *M. tuberculosis *burden countries. Data collected between 2002 and 2007 indicate that the global prevalence of drug-resistant *M. tuberculosis *including MDR vary greatly. The varied drug-resistance patterns make continuous surveillance of drug resistance an essential component of tuberculosis control program.

**Findings:**

*M. tuberculosis *isolates were obtained from consenting adult tuberculosis patients involved in a placebo-controlled study to evaluate the efficacy of multivitamin supplements on response to anti-Tb treatment in Dar es Salaam, Tanzania. Antimicrobial susceptibility testing was done on four antimicrobial agents namely streptomycin, isoniazid, ethambutol and rifampicin. HIV testing and CD4+ T lymphocytes enumeration were also done. A total of 280 *M. tuberculosis *isolates from 191 (68%) males and 89 (32%) female patients with no previous history of anti-tuberculosis treatment exceeding 4 weeks in the previous 12 months were tested. Among these, 133 (47%) patients were HIV seropositive. Fourteen (5.0%) isolates were resistant to any of the anti-tuberculosis drugs. The prevalence of primary resistance was 5.0%, 0.7%, 0.4% and 0% for isoniazid, streptomycin, rifampicin and ethambutol respectively. One isolate (0.4%) was MDR, with resistance to isoniazid, streptomycin and rifampicin.

**Conclusion:**

M. Tb primary resistance rate in a selected population in Dar es Salaam Tanzania is low and efforts should be undertaken to support the Tuberculosis program.

## Background

Tuberculosis (Tb) remains the world's leading cause of death from a single infectious disease and is often the first indicator of human immunodeficiency virus infection [1-4]. The World Health Organization (WHO) has estimated that one third of the world population is infected with *Mycobacterium tuberculosis *(M. Tb) and the estimated incidence of Tb in 2002 was 8.8 million patients of whom 3.9 million had positive smear and/or culture results [4]. Factors associated with the emergence of multi-drug resistant tuberculosis (MDR-Tb) and their effects on the epidemiology of Tb are complex and multifaceted and include inadequate treatment, irregular drug supply, inappropriate regimens and poor patient compliance.

Primary resistance to anti-Tb drugs occurs when a patient is infected with wild type M. Tb which is resistant to anti-Tb drugs. Acquired resistance to anti-Tb drugs occurs when a patient is infected with susceptible forms of M. Tb which become resistant during treatment. Much higher rates of primary resistance have been observed in HIV-infected patients [2]. The WHO/IUATLD Global Project on Drug Resistance surveillance has produced reliable and accurate data on M. Tb resistance [3,4]. MDR in M. Tb has been defined as resistance to at least isoniazid and rifampicin and cure rates of patients with MDR-Tb are low [2]. Hot spots for MDR include States of the former Soviet Union and China but MDR has been reported in several other countries [2,5-8].

The prevalence of resistance to anti-Tb drugs shows marked geographical differences and has important implications on the selection of an appropriate initial treatment regimen. Global data on anti-Tb resistance indicate a median primary resistance to at least one drug of 9.9%; with 6.5% resistance to streptomycin, 1.8% to rifampicin, 1.0% to ethambutol while that of primary MDR was 1.4% [1]. Recent (2002–2007) global WHO data covering 114 countries including Tanzania and 2 special administered regions of China has shown a global prevalence of primary drug-resistant *M. tuberculosis *of between 0% in two Western European countries to 56.3% in Baku, Azerbaijan while the prevalence of MDR ranged from 0% in eight countries to 19.4 and 22.3% in Republic of Moldova and Baku, Azerbaijan respectively [4]. Several studies done in Europe, Middle East and Asia since the late 1990's and early 2000 have shown varied prevalence of resistance to anti Tb drugs [9-12]. Data from several African countries including Malawi, Burundi, S. Africa, Central African Republic and Kenya suggest that M. Tb resistance is of public health importance with an MDR rate ranging from 1.4% to 11.6% [4,6,13-16].

In the era of HIV/AIDS, Tanzania has reported a three-fold increase in notification of Tb and a two-fold increase of new sputum smear-positive tuberculosis [17]. When studying M. Tb isolates collected in Dar es Salaam between 1992 and 1993, Yang et al reported a prevalence of resistance to a single anti-Tb drug of 8.8% while 3.2% of isolates were resistant to more than one drug [18]. In a study by Chum et al, conducted between 1991 and 1993 in Tanzania, the overall prevalence of anti Tb drug resistance was 6.2%, while primary resistance was 4% in HIV-seropositive and 5.8% in HIV-seronegative patients; acquired resistance was 19% [19]. The recently published WHO report has shown that among 369 new M Tb isolates collected in 2007, overall resistance to any of the anti Tb drug was 6.2% while the MDR rate was 1.1% [4]. Here we report on the prevalence of primary resistance of M. Tb to various anti-Tb drugs in Dar es Salaam Tanzania. Tb isolates for this study were collected from HIV seropositive and HIV seronegative patients between 2001 and 2004.

## Methods

Patients attending five of the 14 out-patient Tb treatment clinics in Dar es Salaam, Tanzania were invited to participate in a placebo-controlled study to evaluate the efficacy of multivitamin supplements on response to anti-Tb treatment. Detailed methodology of the study population has been described elsewhere [20,21]. In brief, participants in the trial were men and women aged 18 to 65 years, who had at least two positive sputum smears and who intended to stay in Dar es Salaam for at least 18 months after the completion of Tb therapy. Subjects provided written informed consent to participate in the study. Subjects with Karnofsky scores less than 40% or haemoglobin less than 7.0 gm/dL were excluded from the main study because it was thought that they would not be able to demonstrate significant benefit from the micronutrients. Furthermore, patients with a history of previous Tb treatment exceeding four weeks during the last 12 months or pregnant women were excluded from the study. The sputum samples from patients fulfilling the recruitment criteria were inoculated into Lowenstein Jensen medium and incubated at 37°C for 2–4 weeks. M. Tb colonies were identified based on typical microscopical appearance on Auramine Rhodamine fluorescent stained smears and typical morphology on Lowenstein Jensen medium. Antimicrobial susceptibility testing to isoniazid, streptomycin, rifampicin and ethambutol was done using the proportional method.

Following pretest counselling, blood samples were obtained and tested for presence of HIV antibodies using two sequential ELISAs (Enzygnost anti HIV 1+2 Behring (Marburg, Germany) followed by Wellcozyme Recombinant anti HIV 1 (Murex Biotech Ltd, Dart-ford, UK) in an alternative confirmatory strategy [22]. Samples with discrepant results were retested using Western blot (Genetic System, Redmond, WA), which was interpreted according to WHO criteria [23]. After the results, the patients were post-test counselled and those who were HIV infected were managed according to prevailing national guidelines as anti-retroviral drugs were not available in Tanzania during the study period. Lymphocyte subsets were determined using flow cytometry. Analyses were conducted using the Statistical Package for Social Scientists (SPSS 12.0; Norusis, SPSS Inc., Chicago, IL, USA). Proportions were compared using Chi square tests. Comparison of means was done using unpaired t-test. A two-sided p-value of less than 0.05 was considered as statistically significant.

The study protocol was approved by ethical committees in Muhimbili University of Health and Allied Sciences and Harvard School of Public Health. All patients were treated following national guidelines of management of tuberculosis and HIV infections prevailing during the study period.

### The study population

A total of 887 Tb patients living in Dar es salaam, Tanzania were enrolled in the trial, of which 590 (67%) were males and 297 (33%) females. Among these, 416 (47%) were HIV seronegative while 471 (53%) were HIV-1 seropositive. Of the 887 patients enrolled, sputum culture and sensitivity testing was done on 722 (81%) patients. Among these, sputum culture and sensitivity testing for 280 patients was done between the date of recruitment into the study and one month of starting anti-Tb treatment while for the remaining 442 patients, it was done after the first month of starting anti-Tb treatment. The data from the later group of patients were not included in the current analysis because participants had taken the anti-Tb drugs for some time before sputum collection which can influence the antimicrobial susceptibility pattern of the M Tb isolates. There were no significant differences in the mean age or sex distribution between the patients enrolled in the main study (887), those who had culture results (722) and those who had culture and sensitivity results during the first month of treatment (280). However, the proportion of HIV seropositive patients was significantly lower among participants with culture data compared to the total study population.

### Antimicrobial resistance

Among the 280 M. Tb isolates, 191 (68%) and 89 (32%) were obtained from males and female patients respectively. The mean (median, SD) age of the patients was 32 years (31.0, 8) and 32.0 years (29.0, 9) for males and females respectively (p = 0.48). One hundred forty seven (53%) isolates were obtained from HIV seronegative patients while 133 (47%) were obtained from HIV seropositive patients. Among the HIV seropositive patients, 44 (39%) had CD4+ T lymphocytes ranging from 0 to 199/μL, 47 (42%) from 200–499/μL and 22 (19%) had CD4+ T lymphocytes 500/μL or more. For 20 (15%) patients we did not have CD4 data at recruitment.

Table 1 shows the resistance pattern of the M. Tb isolates. Among the 280 M. Tb isolates tested, 14 were resistant to at least one of the anti-Tb drugs and of these eight isolates were from HIV seropositive patients while 6 were from HIV seronegative patients; four isolates were from females and 10 were from males. The MDR isolate was from an HIV-seropositive patient with 641 CD4+ T lymphocytes/μL living in Dar es Salaam during the study period

**Table 1:** The antimicrobial susceptibility pattern of the M tuberculosis isolates.

	**Resistance to**					
	**Isoniazid**	**Streptomycin**	**Rifampicin**	**Ethambutol**	**Any**	**MDR**

**N**	14/280	2/280	1/280	0	14/280	1/280
**%**	5.0	0.7	0.4	0	5.0	0.4

The primary anti-Tb resistance pattern in the current study is similar to that of another Tanzanian study done between 1991 and 1993 with a primary mono-resistance rate of 0.3% to rifampicin and an MDR prevalence of 0.4% [19]. In addition our findings are similar to the recent primary resistance rates of M Tb isolates collected in Tanzania in 2007 which were reported by WHO [4]. However these resistance rates are low in comparison to the global summary by Pablos-Mendez et al which reported a median primary resistance of 9.9% to any of the anti-Tb drugs and an MDR of 1.4% [1], and are also similar to reports from Burundi, South Africa and Kenya [13,14,16].

Most published M. Tb resistance reports tend to combine results obtained from patients who are treated for the first time and those with history of previous anti Tb treatment. Globally, acquired resistance figures are much higher compared to primary resistance. Our data was based on patients with no history of use of anti-Tb drugs exceeding 4 weeks in the previous 12 months and therefore, may not reflect the prevalence of acquired resistance [1]. There are published reports suggesting that the HIV pandemic and Tb epidemic are concurrently fuelling each other and may be associated with increased resistance to anti-Tb drugs [2]. In the current study there was no evidence pointing to increased resistance in HIV-seropositive patients, probably due to the low prevalence of the resistant strains and also because only primary resistance was analysed as compared to acquired resistance.

One major limitation in the current analysis is the exclusion of patients with a Karnofsky score of less than 40% which resulted in exclusion of patients with more severe disease and possibly those with resistant strains including MDR and X-MDR which is however more common in acquired compared to primary resistance.

## Conclusion

The M. Tb primary resistance rate in this selected population in Dar es Salaam Tanzania is low and efforts should be undertaken to support the Tuberculosis program and conduct further studies in order to guide treatment of M Tb patients in Tanzania.

## Competing interests

The authors declare that they have no competing interests.

## Authors' contributions

Willy Urassa Concept design, conduct of the study, analysis and interpreting of the data, drafting and revision of the paper

Ferdinand Mugusi Concept design, conduct of the study, analysis and interpreting of the data, and revision of the paper

Eduardo Villamor Concept design, conduct of the study, analysis and interpreting of the data, drafting and revision of the paper

Gernard Msamanga Concept design, conduct of the study, drafting and revision of the paper

Candida Moshiro Analysis and interpreting of the data, drafting and revision of the paper

Ronald Bosch Concept design, conduct of the study, analysis and interpreting of the data, drafting and revision of the paper

Elmar Saathoff Conduct of the study, analysis and interpreting of the data and revision of the paper

Wafaie Fawzi Concept design, conduct of the study, analysis and interpreting of the data, drafting and revision of the paper
